# Sources and Amounts of Animal, Dairy, and Plant Protein Intake of US Adults in 2007–2010

**DOI:** 10.3390/nu7085322

**Published:** 2015-08-21

**Authors:** Stefan M. Pasiakos, Sanjiv Agarwal, Harris R. Lieberman, Victor L. Fulgoni

**Affiliations:** 1 Military Nutrition Division, US Army Research Institute of Environmental Medicine, Natick, MA 01760-5007, USA; 2 Oak Ridge Institute for Science and Education, Belcamp, MD 21017, USA; 3 NutriScience LLC, East Norriton, PA 19403, USA; E-Mail: agarwal47@yahoo.com; 4 Military Nutrition Division, US Army Research Institute of Environmental Medicine, Natick, MA 01760-5007, USA; E-Mail: harris.r.lieberman.civ@mail.mil; 5 Henry M. Jackson Foundation, Bethesda, MD 20817, USA; 6 Nutrition Impact LLC, Battle Creek, MI 49014, USA; E-Mail: VIC3RD@aol.com

**Keywords:** dietary guidelines, NHANES, protein density, recommendations, energy intake

## Abstract

Dietary guidelines suggest consuming a mixed-protein diet, consisting of high-quality animal, dairy, and plant-based foods. However, current data on the distribution and the food sources of protein intake in a free-living, representative sample of US adults are not available. Data from the National Health and Nutrition Examination Survey (NHANES), 2007–2010, were used in these analyses (*n* = 10,977, age ≥ 19 years). Several US Department of Agriculture (USDA) databases were used to partition the composition of foods consumed into animal, dairy, or plant components. Mean ± SE animal, dairy, and plant protein intakes were determined and deciles of usual intakes were estimated. The percentages of total protein intake derived from animal, dairy, and plant protein were 46%, 16%, and 30%, respectively; 8% of intake could not be classified. Chicken and beef were the primary food sources of animal protein intake. Cheese, reduced-fat milk, and ice cream/dairy desserts were primary sources of dairy protein intake. Yeast breads, rolls/buns, and nuts/seeds were primary sources of plant protein intake. This study provides baseline data for assessing the effectiveness of public health interventions designed to alter the composition of protein foods consumed by the American public.

## 1. Introduction

Protein is, unquestionably, required in the human diet [1]. Dietary protein is the primary source of amino acids, particularly the essential amino acids, which cannot be synthesized from endogenous precursors, and are required for growth, development, and maintenance of human health. The recommended dietary allowance (RDA) for protein is 0.8 g protein per kilogram body weight (g/kg BW) and is considered adequate for nearly all healthy US adults [1], although consuming protein above the RDA has consistently been shown to be metabolically advantageous by promoting healthy blood lipids, weight management, satiety, and enhancing long-term bone mineralization [2]. We recently reported that habitually consuming a higher protein diet, regardless of body size, was associated with lower adiposity and higher HDL-cholesterol compared to consuming protein at levels consistent with the RDA [3].

The source of dietary protein is perhaps as important as the total quantity consumed. Animal, dairy, and some plant proteins are considered high-quality proteins that confer health and metabolic benefits based on the digestible levels of the essential amino acids they contain. Previous work has shown that many protein foods, regardless if classified as animal, dairy, or plant, are major contributors of other critical nutrients (e.g., zinc, vitamin B-12, iron, calcium, phosphorus, magnesium, vitamin E, and dietary fiber) [4]. The 2010 Dietary Guidelines for Americans recommends consuming a mixed-protein diet, consisting of a variety of high-quality animal, dairy, and plant-based foods [5]. Recently, the 2015 Scientific Report of the Dietary Guidelines Advisory Committee (DGAC) recommended increasing certain plant-based foods, including whole-grains, legumes, and nuts, as well as increasing the intake of low-fat dairy and certain animal-based foods, such as seafood. In contrast, the DGAC recommends consuming a diet lower in plant-based foods that contain refined grains and added sugars and certain animal-based foods, primarily red and processed meat [6]. Whether these recommendations are being met or to what extent dietary modification is required to comply with the DGAC recommendations is not known, largely because no studies have provided comprehensive data of habitual intakes of animal, dairy, and plant-based foods, particularly as they relate to total protein intake, in a representative sample of the free-living US adult population.

The objective of the current study was to determine dietary intake level and food sources of animal, dairy, and plant protein among US adults using data from NHANES 2007–2010. Based on a 2005–2006 characterization of protein intake among older adults using NHANES data [7], we anticipated that animal protein intake, with or without including dairy, would be the predominant source of protein in the diet followed by plant protein. We expected dairy protein intake alone to contribute the lowest amount of total protein to the diet, and that milk, which may provide the highest-quality protein, would be the primary source of dairy protein in the diet.

## 2. Experimental Section

### 2.1. Participants

The study sample consisted of 10,977 adults (age ≥ 19 years) who completed a 24-h dietary recall in *What We Eat in America*, the dietary interview component of the NHANES, 2007–2010. Analyses included only individuals with complete and reliable dietary records using the USDA automated multiple-pass method. Pregnant or lactating women were excluded. All participants or proxies provided written informed consent and the Research Ethics Review Board at the National Center for Health Statistics approved the survey protocol. Detailed description of the survey design and the data collection procedures are reported elsewhere [8].

### 2.2. Estimating Level and Source of Protein Intake

USDA food composition databases were used to determine protein gram intake and protein type from foods consumed by NHANES participants. This process estimates nutrient content of reported foods, by linking the Food and Nutrient Database for Dietary Studies (FNDDS) with food composition data provided by the USDA Nutrient Database for Standard Reference (SR). The ingredients of disaggregated survey food recipes (coded using the SR food codes) were linked to the appropriate food composition databases using the SR-Link file of the FNDDS (versions 4.1 and 5.0 link SR releases 22 and 24 respectively) [9,10].

Protein gram amounts by type associated with an intake in the NHANES individual foods file was obtained via the FNDDS SR Links and SR nutrients files. Every SR code with protein was assigned via the SR code description to a source; animal, dairy, plant or mixed protein. Mixed protein was used to denote that the source for the SR code was from more than one of animal, dairy or plant proteins. For each food code, the SR weights and SR links were used to determine the percentage of protein of each of the types (animal, dairy, plant, mixed) that made up the protein in the food code. These percentages were then applied to the total protein for the food code if each food consumed by each subject. The calculations were done separately for each NHANES data release using the individual food files, FNDDS and SR files corresponding to that NHANES release.

The nutrient profiles for some missing SR codes were obtained from addendums to FNDDS files for missing SR codes. There were only 13 remaining SR codes with missing nutrient profiles. These were obtained from the nearest SR version where it was available or from SR codes with similar descriptions.

An analysis of all the protein intake in the NHANES files indicated these methods result in more than 90% of all protein gram intake categorized into animal, dairy or plant with only less than 10% in the mixed category. An example of protein in the mixed category was pizza. For example, the food code 58106220 “Pizza, cheese, from restaurant or fast food, thin crust” links to the single SR code 21301 denoting pizza. Using the SR description the protein is assigned to the mixed category since its source contains both dairy and plant-based protein and the individual amounts in the dairy and plant categories cannot be calculated from the SR data. Several food categories (such as mixed dishes, burritos and tacos, soups, cakes and pies, and eggs and omelets) were common for more than one source of protein.

Additionally the USDA list of 150 total food categories [11], of which 24 food categories were identified as sources of animal proteins (providing at least 1% animal protein), 20 food categories as sources of dairy protein (providing at least 1% dairy protein), and 31 food categories as sources of plant protein (providing at least 1% of plant protein), was used to define sources of protein by type in the US diet.

### 2.3. Statistical Analysis

Data were analyzed using SAS 9.2 (SAS Institute) and SUDAAN release 11.0 (Research Triangle Institute). Appropriate weighting factors were used to adjust for oversampling of selected groups, survey non-responses of some individuals, and for day of the week the interview was conducted [12]. Mean and percentages ± standard errors (SE) of animal, dairy, and plant protein were determined using PROC DESCRIPT of SUDAAN using data from the first 24-h recall. To develop deciles of animal, dairy, and plant protein intake, individual usual intakes were estimated using the National Cancer Institute (NCI) method [13] similar to that we reported previously [3]. Briefly, usual intake of animal, dairy, and plant protein was estimated using both days of 24-h recall with a single component model since these dietary components are consumed by almost all subjects on most days.

## 3. Results

**Figure 1:**
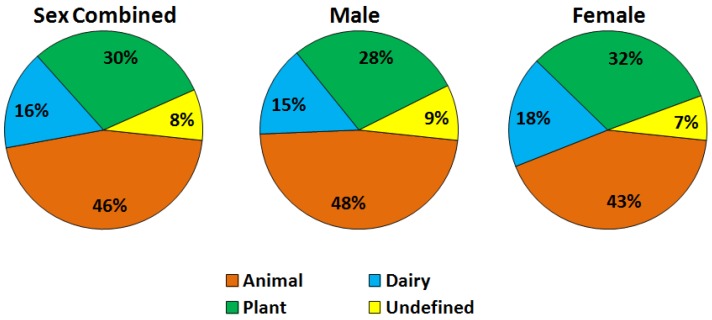
Percentage of animal, dairy, and plant protein intake among US adults combined and separated by sex using data from NHANES 2007–2010 (*n* = 10,977, ≥19 years). Total protein intake (mean ± SE) was 82.3 ± 0.8 g/day (98.6 ± 1.1 g/day for men and 67.0 ± 0.7 g/day for women).

Overall protein intake (mean ± SE) was 82.3 ± 0.8 g/day (98.6 ± 1.1 g/day for men and 67.0 ± 0.7 g/day for women) regardless of protein source among this representative sample of US adults. The proportion of total protein intake attributable to animal protein was 46%, whereas dairy and plant protein accounted for 16% and 30% of total protein intake, respectively (Figure 1). About 8% of the total protein intake (mainly from mixed foods) was undefined because its protein type could not be determined with confidence.

**Table 1:** Demographics of US adults (age 19 years and older) according to absolute (g/day) and relative (g/kg BW) protein intake from different protein source types (NHANES 2007–2010).

	Deciles of Individual Usual Protein Intake									
	D1	D2	D3	D4	D5	D6	D7	D8	D9	D10
**Population with Protein Intake from Animal Sources**										
Sample Size (*n*)	1105	1081	1148	1127	1162	1120	1096	1061	1015	1062
Median animal protein intake (g/kg BW)	0.267	0.343	0.381	0.411	0.441	0.476	0.514	0.554	0.600	0.689
Animal protein intake (g/day)	2.9 ± 0.1	10.7 ± 0.3	20.9 ± 0.3	26.7 ± 0.5	31.3 ± 0.7	35.8 ± 0.7	40.2 ± 0.6	47.1 ± 0.6	61.6 ± 0.9	98.0 ± 1.7
Female (%)	93.0 ± 0.9	78.9 ± 2.4	89.0 ± 1.2	80.4 ± 1.7	65.8 ± 2.1	47.2 ± 2.1	30.4 ± 1.4	13.1 ± 1.6	10.8 ± 1.1	3.4 ± 0.7
Age (years)	49.3 ± 0.9	47.2 ± 0.7	50.0 ± 0.8	50.4 ± 0.6	49.8 ± 0.7	49.4 ± 0.6	47.1 ± 0.6	44.7 ± 0.6	40.7 ± 0.6	37.9 ± 0.5
Ethnicity (%)										
Hispanic	10.6 ± 1.4	11.8 ± 2.0	11.5 ± 2.0	12.8 ± 1.9	13.3 ± 1.7	12.8 ± 2.0	12.3 ± 1.7	14.5 ± 2.2	15.8 ± 2.1	20.2 ± 2.6
White	75.3 ± 2.7	71.8 ± 3.0	71.3 ± 2.6	72.1 ± 3.2	70.0 ± 2.7	69.4 ± 3.0	69.1 ± 3.3	68.2 ± 2.9	65.1 ± 2.8	61.0 ± 3.4
Black	8.9 ± 1.2	10.0 ± 1.3	13.2 ± 1.7	11.5 ± 1.7	12.0 ± 1.6	10.9 ± 1.3	11.6 ± 1.5	11.5 ± 1.4	12.2 ± 1.4	12.1 ± 1.2
Other	5.2 ± 1.0	6.4 ± 1.3	3.9 ± 0.8	3.6 ± 0.9	4.7 ± 0.9	6.9 ± 1.4	6.9 ± 1.4	5.8 ± 1.6	6.9 ± 1.0	6.7 ± 1.5
**Population with Protein Intake from Dairy Sources**										
Sample Size (*n*)	1258	1313	1177	1105	1055	1134	1042	1023	940	930
Median dairy protein intake (g/kg BW)	0.070	0.105	0.133	0.150	0.165	0.179	0.195	0.212	0.235	0.279
Dairy protein intake (g/day)	0.03 ± 0.003	1.2 ± 0.04	4.0 ± 0.1	6.4 ± 0.1	8.9 ± 0.1	11.7 ± 0.2	15.1 ± 0.2	19.0 ± 0.2	25.1 ± 0.3	41.7 ± 0.9
Female (%)	48.5 ± 1.7	54.3 ± 2.2	54.4 ± 1.6	55.9 ± 1.5	52.1 ± 1.9	53.6 ± 1.8	47.3 ± 1.9	49.0 ± 1.6	51.4 ± 1.9	45.3 ± 1.9
Age (years)	44.5 ± 0.7	48.2 ± 0.7	48.2 ± 0.7	47.9 ± 0.7	46.7 ± 0.8	48.3 ± 0.8	46.4 ± 0.7	46.6 ± 0.6	45.0 ± 0.8	44.6 ± 0.7
Ethnicity (%)										
Hispanic	18.9 ± 2.7	14.8 ± 2.4	13.1 ± 2.1	13.1 ± 2.0	12.4 ± 2.1	12.6 ± 1.6	14.8 ± 1.8	12.5 ± 2.0	12.2 ± 1.8	11.2 ± 1.7
White	51.5 ± 3.9	58.9 ± 3.6	65.3 ± 3.3	66.5 ± 3.5	73.1 ± 3.0	72.3 ± 2.4	71.3 ± 2.7	75.0 ± 2.7	79.3 ± 2.5	80.4 ± 2.1
Black	20.3 ± 2.2	19.1 ± 2.2	14.8 ± 1.8	13.5 ± 1.6	10.4 ± 1.4	9.0 ± 1.1	8.4 ± 1.2	8.8 ± 1.1	5.2 ± 0.8	4.4 ± 0.6
Other	9.4 ± 1.8	7.2 ± 1.5	6.8 ± 1.5	6.9 ± 1.5	4.1 ± 0.8	6.1 ± 1.1	5.4 ± 1.1	3.8 ± 0.8	3.4 ± 0.9	3.9 ± 0.9
**Population with Protein Intake from Plant Sources**										
Sample Size (*n*)	1203	1174	1133	1116	1104	1011	1054	1067	1042	1073
Median plant protein intake (g/kg BW)	0.205	0.240	0.263	0.281	0.298	0.316	0.334	0.358	0.389	0.453
Plant protein intake (g/day)	7.6 ± 0.2	12.6 ± 0.1	16.2 ± 0.1	18.7 ± 0.1	21.5 ± 0.3	23.8 ± 0.2	27.1 ± 0.2	30.6 ± 0.3	36.0 ± 0.2	52.1 ± 0.6
Female (%)	67.3 ± 1.8	61.8 ± 1.5	59.6 ± 2.2	56.8 ± 2.2	51.0 ± 2.3	48.8 ± 2.5	48.5 ± 2.4	39.9 ± 1.8	43.4 ± 1.9	34.7 ± 1.9
Age (years)	43.8 ± 0.6	46.5 ± 0.7	48.9 ± 0.5	49.0 ± 0.8	48.9 ± 0.5	47.2 ± 0.6	46.7 ± 0.8	46.5 ± 0.7	46.3 ± 0.9	42.6 ± 0.7
Ethnicity (%)										
Hispanic	12.5 ± 2.1	14.2 ± 2.2	10.4 ± 1.5	13.6 ± 1.9	11.5 ± 1.9	11.5 ± 1.7	13.5 ± 1.7	15.0 ± 2.5	15.8 ± 2.2	17.7 ± 2.5
White	62.0 ± 3.6	66.2 ± 3.2	71.8 ± 2.8	71.4 ± 3.1	71.4 ± 2.9	73.8 ± 2.5	69.1 ± 2.9	69.7 ± 3.3	69.9 ± 3.0	68.2 ± 3.3
Black	22.0 ± 2.5	15.8 ± 2.2	13.2 ± 1.6	11.6 ± 1.7	10.6 ± 1.4	9.8 ± 1.2	9.3 ± 1.3	8.1 ± 1.0	6.6 ± 0.8	6.9 ± 0.9
Other	3.5 ± 0.8	3.8 ± 0.8	4.6 ± 1.0	3.4 ± 0.9	6.5 ± 1.4	5.0 ± 1.1	8.1 ± 1.6	7.2 ± 1.2	7.8 ± 1.9	7.1 ± 1.4

Animal, dairy, and plant protein intake more than doubled from population decile 1 to decile 10 (Table 1). Animal protein intake was 2.6-fold and dairy protein intake four-fold higher in decile 10 compared to decile 1. Plant protein intake was 2.2-fold higher in decile 10 compared to decile 1. More than half of the women surveyed reported consuming levels of animal and plant protein in deciles 1 through 5, whereas the proportion of women consuming dairy protein was relatively constant across all deciles. The percentage of White individuals decreased and Hispanic individuals increased across deciles of protein intake from animal foods. However, the percentage of Whites reporting consuming more dairy protein increased across deciles, whereas the percentage of Hispanics and Blacks decreased across deciles. More Hispanics tended to consume higher levels of plant protein, while a greater percentage of Blacks reported consuming lower levels of plant protein (Table 1).

Twenty-four food categories were identified as contributing at least 1% of total animal protein intake. Chicken and beef were the top two food categories for animal protein, providing 26% of total animal protein intake, 13% of total dietary protein intake, and 5% of total energy intake. The top 10 animal protein food categories, each of which contributed to more than 3% of total animal protein intake, provided approximately 67% of total animal protein intake but less than 16% of total energy intake (Table 2).

**Table 2:** Food sources of animal protein (providing at least 1% animal protein) and energy among US adults age 19 years and older. Data from NHANES 2007–2010.

Food Categories	Animal Protein		Total Protein		Total Energy	
	Rank	% Total	Rank	%Total	Rank	% Total
Chicken, whole pieces	1	13.9 ± 0.5	1	7.2 ± 0.3	2	2.8 ± 0.1
Cold cuts and cured meats	2	9.2 ± 0.2	5	3.6 ± 0.1	9	1.3 ± 0.04
Meat mixed dishes *	3	7.3 ± 0.3	2	3.9 ± 0.2	4	2.0 ± 0.1
Eggs and omelets *	4	7.2 ± 0.3	6	3.3 ± 0.1	5	1.9 ± 0.1
Beef, excludes ground	5	6.9 ± 0.3	4	3.6 ± 0.2	8	1.4 ± 0.1
Ground beef	6	5.6 ± 0.4	8	2.6 ± 0.2	12	1.0 ± 0.1
Fish	7	5.0 ± 0.3	9	2.5 ± 0.2	13	1.0 ± 0.1
Poultry mixed dishes *	8	4.8 ± 0.3	7	2.7 ± 0.2	7	1.5 ± 0.1
Pork	9	4.5 ± 0.3	12	2.3 ± 0.2	14	0.9 ± 0.1
Soups *	10	3.1 ± 0.3	10	2.5 ± 0.2	6	1.7 ± 0.1
Seafood mixed dishes	11	2.6 ± 0.3	16	1.2 ± 0.1	18	0.6 ± 0.1
Pasta mixed dishes, excludes macaroni and cheese *	12	2.4 ± 0.2	11	2.4 ± 0.1	3	2.1 ± 0.1
Sausages	13	2.2 ± 0.2	18	1.0 ± 0.1	16	0.7 ± 0.1
Frankfurters	14	2.1 ± 0.2	20	0.8 ± 0.1	19	0.6 ± 0.1
Pizza	15	2.0 ± 0.2	3	3.8 ± 0.2	1	3.1 ± 0.1
Stir-fry and soy-based sauce mixtures	16	2.0 ± 0.2	15	1.3 ± 0.1	17	0.7 ± 0.1
Turkey, duck, other poultry	17	1.9 ± 0.2	19	0.9 ± 0.1	23	0.3 ± 0.04
Bacon	18	1.7 ± 0.1	23	0.7 ± 0.04	22	0.4 ± 0.02
Chicken/turkey sandwiches	19	1.4 ± 0.2	17	1.1 ± 0.1	15	0.8 ± 0.1
Burritos and tacos *	20	1.4 ± 0.1	14	1.5 ± 0.1	11	1.1 ± 0.1
Shellfish	21	1.4 ± 0.1	24	0.7 ± 0.1	24	0.3 ± 0.03
Other sandwiches	22	1.1 ± 0.1	21	0.7 ± 0.1	20	0.6 ± 0.04
Other Mexican mixed dishes	23	1.1 ± 0.1	22	0.7 ± 0.1	21	0.5 ± 0.1
Burgers	24	1.0 ± 0.2	13	1.7 ± 0.2	10	1.1 ± 0.1

* These food categories also contribute as dairy and/or plant protein food sources and are included in Table 3 and/or Table 4.

Twenty food categories were identified as providing at least 1% of dairy protein intake. Cheese and reduced fat milk were the top two food categories for dairy protein, providing approximately 35% of total dairy protein intake, 6% of total protein intake, and 4% of total energy intake (Table 3). Reduced fat, nonfat, whole, and low fat milk combined provided approximately 28% of total dairy protein intake, 6% of total protein intake, and 3% of total energy intake. The top 10 dairy protein food categories provided nearly 70% of total dairy protein intake and 11% of total energy intake.

**Table 3:** Food sources of dairy protein (providing at least 1% dairy protein) and energy among US adults age 19 years and older. Data from NHANES 2007–2010.

Food Categories	Dairy Protein		Total Protein		Total Energy	
	Rank	% Total	Rank	%Total	Rank	% Total
Cheese	1	23.4 ± 0.6	1	4.3 ± 0.2	1	2.6 ± 0.1
Milk, reduced fat	2	11.3 ± 0.5	5	2.2 ± 0.1	8	1.3 ± 0.1
Ice cream and frozen dairy desserts	3	6.4 ± 0.3	8	1.0 ± 0.04	4	1.9 ± 0.1
Milk, nonfat	4	6.4 ± 0.4	7	1.3 ± 0.1	15	0.6 ± 0.04
Milk, whole	5	5.8 ± 0.3	9	1.0 ± 0.1	12	0.7 ± 0.04
Milk, low fat	6	4.7 ± 0.3	11	1.0 ± 0.1	17	0.5 ± 0.05
Cream and cream substitutes	7	3.7 ± 0.2	19	0.2 ± 0.01	13	0.6 ± 0.02
Eggs and omelets *	8	3.4 ± 0.2	2	3.3 ± 0.1	5	1.9 ± 0.1
Yogurt, low fat and nonfat	9	3.3 ± 0.2	15	0.7 ± 0.03	16	0.5 ± 0.03
Coffee *	10	2.1 ± 0.2	10	1.0 ± 0.1	14	0.6 ± 0.03
Macaroni and cheese	11	2.0 ± 0.3	12	1.0 ± 0.1	11	0.8 ± 0.11
Mashed potatoes and white potato mixtures *	12	1.8 ± 0.2	16	0.6 ± 0.03	10	1.0 ± 0.1
Pasta mixed dishes, excludes macaroni and cheese *	13	1.8 ± 0.2	4	2.4 ± 0.1	3	2.1 ± 0.1
Burritos and tacos *	14	1.3 ± 0.1	6	1.5 ± 0.1	9	1.1 ± 0.1
Cream cheese, sour cream, whipped cream	15	1.3 ± 0.2	20	0.1 ± 0.01	19	0.3 ± 0.02
Cottage/ricotta cheese	16	1.2 ± 0.1	18	0.4 ± 0.04	20	0.1 ± 0.01
Dips, gravies, other sauces	17	1.2 ± 0.1	17	0.4 ± 0.03	18	0.4 ± 0.03
Poultry mixed dishes *	18	1.1 ± 0.1	3	2.7 ± 0.2	6	1.5 ± 0.1
Cakes and pies *	19	1.1 ± 0.1	13	0.8 ± 0.04	2	2.2 ± 0.10
Biscuits, muffins, quick breads *	20	1.1 ± 0.1	14	0.7 ± 0.06	7	1.3 ± 0.1

* These food categories also contribute as animal and/or plant protein food sources and are included in Table 2 and/or Table 4.

Thirty-one food categories were identified as providing at least 1% plant protein intake. These 31 plant protein sources provided nearly 73% of plant protein in the diet. Yeast breads and rolls/buns were the top two plant protein food categories, providing nearly 18% of total plant protein intake, 6% total protein and total energy intake. The top 10 plant protein food categories provided approximately 40% of total plant protein intake and 20% of total energy intake (Table 4).

**Table 4:** Food sources of plant protein (providing at least 1% dairy protein) and energy among US adults age 19 years and older. Data from NHANES 2007–2010.

Food Categories	Plant Protein		Total Protein		Total Energy	
	Rank	% Total	Rank	%Total	Rank	% Total
Yeast breads	1	11.6 ± 0.3	2	3.9 ± 0.1	1	4.0 ± 0.1
Rolls and buns	2	5.9 ± 0.3	7	1.7 ± 0.1	9	1.9 ± 0.1
Nuts and seeds	3	4.7 ± 0.2	6	2.0 ± 0.1	6	2.0 ± 0.1
Pasta mixed dishes, excludes macaroni and cheese *	4	3.1 ± 0.2	5	2.4 ± 0.1	4	2.1 ± 0.1
Beans, peas, legumes	5	2.9 ± 0.2	9	1.3 ± 0.1	23	1.0 ± 0.1
French fries and other fried white potatoes	6	2.6 ± 0.1	13	0.8 ± 0.04	8	2.0 ± 0.1
Tortillas	7	2.5 ± 0.27	11	0.9 ± 0.1	17	1.2 ± 0.1
Beer	8	2.3 ± 0.12	18	0.7 ± 0.04	3	2.2 ± 0.1
Cookies and brownies	9	2.1 ± 0.1	16	0.7 ± 0.02	7	2.0 ± 0.1
Ready-to-eat cereal, higher sugar (>21.2 g/100 g)	10	2.1 ± 0.1	19	0.7 ± 0.04	16	1.2 ± 0.1
Ready-to-eat cereal, lower sugar (≤21.2 g/100 g)	11	2.1 ± 0.1	15	0.7 ± 0.05	22	1.0 ± 0.1
Rice	12	2.1 ± 0.2	17	0.7 ± 0.1	15	1.3 ± 0.1
Coffee *	13	2.1 ± 0.1	10	1.0 ± 0.1	29	0.6 ± 0.03
Doughnuts, sweet rolls, pastries	14	2.0 ± 0.1	20	0.7 ± 0.04	12	1.4 ± 0.1
Biscuits, muffins, quick breads *	15	1.9 ± 0.1	14	0.7 ± 0.1	13	1.3 ± 0.1
Bagels and English muffins	16	1.8 ± 0.1	21	0.7 ± 0.05	28	0.6 ± 0.05
Cakes and pies *	17	1.7 ± 0.1	12	0.8 ± 0.04	2	2.2 ± 0.1
Candy containing chocolate	18	1.7 ± 0.1	25	0.6 ± 0.04	14	1.3 ± 0.1
Soups *	19	1.7 ± 0.1	4	2.5 ± 0.2	10	1.7 ± 0.1
Meat mixed dishes *	20	1.6 ± 0.1	1	3.9 ± 0.2	5	2.0 ± 0.1
Tortilla, corn, other chips	21	1.5 ± 0.1	26	0.5 ± 0.03	18	1.2 ± 0.1
Mashed potatoes and white potato mixtures *	22	1.4 ± 0.1	23	0.6 ± 0.03	21	1.0 ± 0.1
Crackers, excludes saltines	23	1.4 ± 0.1	27	0.5 ± 0.03	25	0.8 ± 0.04
Other vegetables and combinations	24	1.4 ± 0.1	29	0.5 ± 0.03	31	0.5 ± 0.03
Potato chips	25	1.4 ± 0.1	31	0.4 ± 0.02	20	1.1 ± 0.1
Oatmeal	26	1.4 ± 0.1	24	0.6 ± 0.03	26	0.7 ± 0.04
Poultry mixed dishes *	27	1.3 ± 0.1	3	2.7 ± 0.2	11	1.5 ± 0.1
Citrus juice	28	1.2 ± 0.1	30	0.4 ± 0.03	24	1.0 ± 0.04
Rice mixed dishes	29	1.2 ± 0.1	22	0.6 ± 0.1	27	0.7 ± 0.1
Pancakes, waffles, French toast	30	1.1 ± 0.1	28	0.5 ± 0.03	30	0.5 ± 0.04
Burritos and tacos *	31	1.0 ± 0.1	8	1.5 ± 0.1	19	1.1 ± 0.1

* These food categories also contribute as animal and/or dairy protein food sources and are included in Table 2 and/or Table 3.

**Figure 2:**
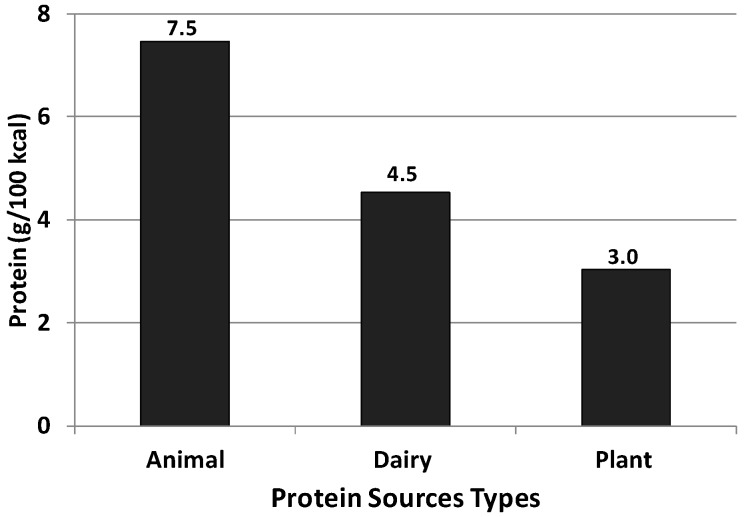
Protein density of animal protein foods *, dairy protein foods *, and plant protein foods *. (* Providing at least 1% animal, dairy or plant protein).

The protein density (g/100 kcal) of animal protein food sources (providing at least 1% animal protein) was more than two times the density of plant protein food sources (providing at least 1% plant protein) and 50% more than dairy protein food sources (providing at least 1% dairy protein). The protein density of dairy protein food categories was also 50% more than the density of plant protein food categories (Figure 2).

## 4. Discussion

The primary finding from this cross-sectional study confirms that Americans habitually consume protein that is predominately animal-based. Chicken, a lean source of high-quality protein, and beef were the primary source of animal protein intake. Plant protein did account for more than a third of total protein intake, although the major food sources of plant protein intake were breads and thus not plant foods that contain generally high-quality sources of protein. The proportion of total protein intake attributed to dairy (16%) was relatively low in comparison to animal (46%) and plant (30%) protein intake, although the primary sources of dairy protein intake in the American adult diet (e.g., cheese and milk) are considered to be among the highest-quality sources of protein.

Although our estimates of total protein intake are consistent with our previous reports [3,14], few studies using national representative data have systematically characterized the level and also the food sources of animal, dairy, and plant protein intake in the American diet. Not surprisingly, those studies reported that animal-derived protein (including dairy) was the main source of protein intake. Analyses of data from NHANES [7,15] and European adults [16,17] show animal and dairy protein intake accounted for more than two thirds of total protein intake. Their findings are consistent with the combined total intake of protein from animal and dairy-based sources we found. Plant protein accounted for nearly one third of total dietary protein intake in our study, primarily from grains, which were also the primary source of plant protein intake among US adults nearly two [18] and three decades ago [15]. These data suggest American dietary habits are, essentially, unchanged, despite official national policy recommendations [19] promoting substantial changes and the popularity of various diets (e.g., the Paleo, Atkins, Gluten-free, South Beach, DASH, *etc.*).

Food sources of animal protein were the most efficient source of dietary protein compared to food sources of dairy and plant-based protein when expressed as protein density (*i.e.*, the amount of protein per 100 kcal). Animal protein foods were twice as dense as plant protein foods. Dairy protein foods were also more protein-dense than plant protein foods. Animal and dairy proteins are considered high-quality proteins and excellent sources of other required nutrients, including iron, calcium, and vitamin D [20]. Plant protein foods are generally less protein-dense and, as a consequence, their consumption results in intake of more energy relative to protein. Grains, most of which were likely refined grains and foods with added sugar, were the largest contributor to total plant protein intake. Grains are often considered incomplete proteins because the essential amino acid content is low, with lysine as the most deficient [21]. Perhaps most importantly, the number of plant-based food categories contributing to total plant protein intake are, essentially, the same types of foods (e.g., doughnuts, cakes, pies, biscuits, candy, *etc.*) that the DGAC [6] recommends should be consumed less in order to achieve a healthier diet. Overconsumption of these foods likely diminishes the quality of total protein intake at the expense of simply consuming more energy.

Our sample size (10,977), consistent approach to classifying protein type, and use of the NCI usual intake methodology to estimate usual intakes are strengths of the current study. However, there are some limitations, particularly reliance on self-report dietary recalls, which can under and overestimate actual intake data [22], even though NHANES uses sophisticated interview procedures. We acknowledge the timeframe assessed (NHANES 2007–2010) may not entirely reflect current intakes and that our analytical approach was unable to differentiate 100% of the protein in the diet, although we were able to confidently differentiate more than 90% of protein intake as animal, dairy, and plant.

## 5. Conclusions

This population-based, descriptive study provides a comprehensive, contemporary analysis of total level and food sources of animal, diary, and plant protein intake in a representative sample of US adults. Our analyses indicate that, in the American adult diet, animal protein is the predominant source of dietary protein followed by plant and dairy protein. These data demonstrate that in a representative sample of the American adult population about 30% of protein is coming from plant-based foods. However, most of these foods are relatively low in protein density and any dietary recommendation that recommends a diet higher in plant-based foods should consider their effects on energy intake and the quantity and quality of protein consumed, and the positive and negative impact on intake of nutrients associated with these protein-containing foods.
